# Ataxia-Telangiectasia Mutated Kinase: Role in Myocardial Remodeling

**Published:** 2016-12-16

**Authors:** Patsy Thrasher, Mahipal Singh, Krishna Singh

**Affiliations:** 1 Department of Biomedical Sciences, James H Quillen College of Medicine, East Tennessee State University, Johnson City, TN, USA; 2 James H Quillen Veterans Affairs Medical Center, Mountain Home, TN, USA

**Keywords:** Ataxia-telangiectasia mutated kinase, Myocardial remodeling, Apoptosis, β-adrenergic receptor, Myocardial infarction, Fibrosis, Hypertrophy

## Abstract

Ataxia-telangiectasia mutated kinase (ATM) is a serine/threonine kinase. Mutations in the ATM gene cause a rare autosomal multisystemic disease known as Ataxia-telangiectasia (AT). Individuals with mutations in both copies of the ATM gene suffer from increased susceptibility to ionizing radiation, predisposition to cancer, insulin resistance, immune deficiency, and premature aging. Patients with one mutated allele make-up ~1.4 to 2% of the general population. These individuals are spared from most of the symptoms of the disease. However, they are predisposed to developing cancer or ischemic heart disease, and die 7–8 years earlier than the non-carriers. DNA double-strand breaks activate ATM, and active ATM is known to phosphorylate an extensive array of proteins involved in cell cycle arrest, DNA repair, and apoptosis. The importance of ATM in the regulation of DNA damage response signaling is fairly well-established. This review summarizes the role of ATM in the heart, specifically in cardiac remodeling following β-adrenergic receptor stimulation and myocardial infarction.

## Introduction

Ataxia-telangiectasia mutated kinase (ATM), a ~370 kDa serine/threonine kinase, is a member of phosphatidylinositol 3-kinase (PI3K) family of proteins^1–5^. ATM resides primarily in the nucleus^6^, where its main function is to control the cell cycle progression following DNA damage, particularly double strand breaks^7,8^. Once DNA damage occurs, ATM is activated and recruited to DNA double strand breaks. Active ATM phosphorylates downstream targets like Chk2^7^ and p53^9^. Although ATM has mostly been reported as a nuclear protein involved in signaling pathways that control cell cycle and DNA damage recognition, it is also shown to be located in the cytoplasm^1,10^. The N-terminal domain of ATM is suggested to interact with β-adaptin in cytoplasmic vesicles^11^, thereby playing a role in intracellular vesicles and/or protein transport mechanisms.

Mutations in ATM cause a multisystemic disease known as Ataxia-telangiectasia (AT)^1,2,5^. AT, a rare immunodeficiency disorder, is characterized by a plethora of phenotypes including growth retardation, cancer susceptibility, immunodeficiency, cerebellar ataxia, γ-radiation hypersensitivity, insulin resistance, etc.^1–5,12,13^. AT heterozygotes, individuals with an ATM mutation in one allele, make up a substantial portion of the population (~1.4 to 2%)^14,15^. These individuals are spared from most of the symptoms of AT, but are more susceptible to cancer and ischemic heart disease^14,15^. According to the National Center for Health Statistics, over 600,000 people died from heart disease in 2014 in the United States^16^. Heart disease can occur in response to many conditions like coronary artery disease, chronic hypertension, atherosclerosis, and myocardial infarction (MI) ^17^. Although the role of ATM in DNA damage signaling has been extensively studied, little is known about its role in the heart and myocardial remodeling. This review summarizes ATM expression in the heart, and its role in maintenance of basal heart function and myocardial remodeling, with a particular emphasis on the role of ATM in cardiac remodeling following β-adrenergic receptor (β-AR) stimulation and MI.

## ATM: a protein with multiple functions

In addition to being a key sensor of DNA double-stranded break signaling, ATM is also a major player in regulating responses to other genotoxic stresses that ultimately influence pathological outcomes. Evidence suggests the involvement of ATM in several cytoplasmic metabolic pathways. Yang and Kastan provided evidence that ATM promotes protein synthesis by inhibiting the translation repressor 4EBP1 in response to insulin, suggesting that a defect in this pathway may explain the insulin resistance observed in many AT patients^1^. ATM also plays a role in oxidative stress. In fact, reactive oxygen species (ROS) can directly activate ATM^18^. ATM-deficient cells have increased ROS, a factor that may play a role in the development of the neurological phenotypes seen in AT patients^19^. ATM-deficient cells exhibit mitochondrial dysfunction, suggesting a role for ATM in the regulation of mitophagy. ATM-deficient cells also exhibit increased mitochondrial numbers, a feature attributed to impaired mitophagy^20^. These studies suggest that ATM is versatile, playing a role in both maintaining genomic stability and modulating cellular responses to genotoxic stresses.

## ATM: cell survival or apoptosis

In addition to controlling cellular responses to genotoxic stress, ATM also regulates cell fate decisions following genotoxic stress. Exposure to genotoxic stresses can cause cell death via apoptosis. ATM is shown to serve either pro-apoptotic or pro-survival purposes. In AT lymphoid cells, ATM deficiency prevents TRAIL and Fas-induced apoptosis via up-regulation of anti-apoptotic FLIP protein^21^. However, inhibition of ATM activity heightens TRAIL-induced apoptosis in human melanoma cells^22^. In addition, ATM promotes apoptosis in response to overexpression of oncogene *c-myc* in squamous epithelial tissues via p53 activation^23^. However, ATM is not required for p53 activation and apoptosis in epithelial tissues following ionizing radiation^24^. Although ATM plays a role in initiating apoptosis, ATM also activates proteins like NF-κB^25^ and Akt^26^ that enhance the expression of anti-apoptotic genes. Pharmacological inhibition of ATM has been shown to suppress Akt-mediated pro-survival signaling in cancer cells^26^. Furthermore, loss of ATM results in defective proliferation and self-renewal in neuronal stem cells^27^.

## ATM and the heart

### ATM expression

ATM expression increases in response to many genotoxic agents, insulin, oxidative stress, etc. Furthermore, hypoxia and hypothermia increase ATM activation^28,29^. Using gene array technique, our laboratory identified increased expression of ATM in the heart following β-AR stimulation. RT-PCR analysis of total RNA uncovered a 2.5 fold increase in ATM mRNA following β-AR stimulation^30^. The ATM promotor has several *cis-*regulatory sequences, one being a modified AP-1 site (fat specific element; Fse)^31^. The Fse site is an alternate binding site for the AP-1 transcription factor due to its interaction with Fos-Jun complexes^32^. Interestingly, Fos and Jun expression is primarily modulated via ERK1/2 and JNK activation^33^. Thus, it is possible that ERK1/2 and JNK are involved in heightened ATM expression following β-AR stimulation. Myocardial infarction (MI) also increases ATM expression, as evidenced by increased ATM protein levels in the non-infarct and infarct regions of ATM wildtype (WT) and heterozygous knockout (hKO) hearts 1 and 3 days post-MI^34^. This increase in ATM could be due to enhanced sympathetic nerve activity since myocardial ischemia associates with release of catecholamines from sympathetic nerve endings^35^.

### Role in myocardial function

In addition to its role in genotoxic stress responses, ATM plays a role in ventricular function at basal levels and in remodeling. In a study investigating basal structure and function of the heart in ATM knockout (KO) mice, m-mode echocardiography revealed that mice lacking ATM exhibit decreased LV diameters and volumes with no changes in percent fractional shortening (%FS) and ejection fraction (EF)^36^. Myocyte hypertrophy and fibrosis have been identified as key components in regulating heart function^37^. Myocyte hypertrophy provides a compensatory mechanism that improves heart function in response to hemodynamic overload^37^. In this particular study ATM KO mice exhibited enhanced fibrosis and myocyte hypertrophy compared to their WT counterparts^36^. Thus, it is possible that myocyte hypertrophy maintains EF and %FS in the face of increasing fibrosis. Together, these results suggest diastolic impairment in the absence of ATM at basal levels.

As stated previously, heart disease can occur in response to many stimuli, one being MI. Following MI, the heart undergoes cardiac remodeling, a process that ultimately results in cardiac dysfunction^37^. Studies investigating the role of ATM in cardiac remodeling revealed that ATM deficiency has different effects on myocardial remodeling early and late post-MI. Echocardiography revealed that ATM deficient (heterozygous knockout; hKO) mice have higher %FS and EF 1 and 7 days post-MI coupled with lower LV diameters and volumes 1, 3, and 7 days post-MI^34,38^. Interestingly, ATM deficiency also resulted in increased fibrosis and alpha-smooth muscle actin (α-SMA; myofibroblast differentiation marker) expression 3 and 7 days post-MI in addition to an increase in infarct thickness 7 days post-MI^34,38^. Together these results indicate that ATM deficiency reduces functional impairment of the heart early post-MI. Although sustained fibrosis is associated with heart failure, early fibrosis may play a protective role in the healing process by preventing infarct expansion^39^. Myofibroblasts are primarily responsible for fibrosis deposition following MI^39^ and may contribute to the observed infarct thickness in ATM deficient mice, which could aid in attenuating LV dysfunction.

Conversely, another study revealed that ATM deficiency results in heightened LV dysfunction late post-MI as evidenced by decreased %FS and EF% coupled with increased LVESV in ATM deficient mice 14 and 28 days post-MI. ATM deficient mice experienced increased myocyte apoptosis, hypertrophy, and fibrosis post-MI^40^, all factors that contribute to heart failure late post-MI. Interestingly, LV dysfunction is also exacerbated 28 days post β-AR stimulation in ATM deficient mice as measured by decreased %FS and EF%. Such dysfunction is also accompanied by sustained increase in myocyte apoptosis and fibrosis^30^. Collectively, these studies provide evidence that ATM has the potential to affect different phases of cardiac remodeling post-MI. ATM deficiency may play a protective role in the heart early post-injury, but is detrimental late post-injury. Infarct healing early post-MI may benefit from ATM deficiency in terms of function. However, increased hypertrophy, apoptosis and fibrosis may ultimately associate with negative consequences in terms of cardiac function. Future investigations identifying the molecular signals in modulating cell survival and growth during ATM deficiency may help explain the role of ATM in the healing process of the heart post-MI.

### Role in myocyte apoptosis and myocardial fibrosis

Myocytes are the fundamental contractile cell of the myocardium^41^ and play a critical role in cardiac remodeling^42,43^. Thus, myocyte apoptosis is considered to be a determinant of structure and function of the heart, particularly during remodeling^42,43^. p53, a tumor suppressor gene, is a key player in apoptosis and is phosphorylated and stabilized by ATM^9^. Once activated, p53 stimulates the expression of pro-apoptotic genes like Bax^44^. Novel studies investigating the role of ATM in myocyte apoptosis following β-AR stimulation revealed interesting differences between ATM deficient and KO hearts. ATM deficiency (ATM heterozygosity) results in increased myocyte apoptosis 28 days following β-AR stimulation^30^. Here, β-AR stimulation increased expression and phosphorylation of p53 and expression of Bax to a similar extent in WT and hKO hearts. ^30^ However, ATM deficient hearts exhibited reduced levels of β1 integrin (a protective signaling pathway). On the other hand, 24 h β-AR stimulation increased myocyte apoptosis to a similar extent in WT and KO hearts. Here, activation of JNKs and expression and phosphorylation of p53 was only observed in WT hearts in response to β-AR stimulation. However, activation of Akt was lower in KO-sham hearts and remained lower in the KO hearts following β-AR stimulation^36^. Collectively, these studies provide evidence for the involvement of differential signaling pathways leading to apoptosis in ATM deficient and KO hearts in response to β-AR stimulation. Furthermore, β-AR-stimulated apoptosis in WT hearts may involve p53- and JNK-dependent pathways, while apoptosis may be regulated by Akt-mediated pathways in KO hearts. Involvement of Akt signaling mechanism is further supported by the observation of decreased Akt activation in ATM deficient hearts 1 day post-MI. Coincidently, ATM deficiency associated with enhanced activation of GSK-3β (a pro-apoptotic kinase) in those hearts as well^34^. However, β-AR stimulation has no effect on GSK-3β activation in ATM KO hearts^36^, suggesting that ATM mediated apoptosis may primarily be regulated via Akt pathways, while GSK-3β may be activated during particular stress in ATM deficient hearts.

In addition to apoptosis, fibrosis is another common characteristic of heart failure and mostly stems from the production of extracellular matrix (ECM) proteins^39^. Fibroblasts produce matrix metalloproteinases (MMPs) and tissue inhibitors of matrix metalloproteinases (TIMPs), proteinases that play a role in the deposition of fibrosis in the heart^45^. ATM deficiency resulted in increased fibrosis and MMP-2 protein levels under basal conditions and following β-AR stimulation^30,36^. Additionally, TIMP-2 (MMP-2 inhibitor) decreases during ATM deficiency in response to β-AR stimulation^30^. On the contrary, MMP-9 protein levels were higher in the infarct region of ATM deficient hearts 7 days post-MI^38^. MMP-9 expression was also lower in the non-infarct region of ATM deficient hearts 28 days post-MI coupled with an increase in fibrosis^40^. Collectively, these results suggest that MMP-2 plays a role in fibrosis during ATM deficiency at basal levels and following β-AR stimulation. However, MMP-9 may play a role in fibrosis following MI. Changes in MMP-2 and MMP-9 may influence LV remodeling during ATM deficiency and may account for functional changes associated with ATM deficiency.

### Role in myocardial inflammation

MI results in cardiac cell death, thus initiating an intense inflammatory response essential for cardiac repair and remodeling. The post-MI repair process begins with the flood of neutrophils into the infarcted area that remove dead cells via phagocytosis^17^. Macrophages then engulf apoptotic neutrophils, producing anti-inflammatory cytokines like transforming growth factor β (TGF-β). TGF-β is involved in myofibroblast differentiation post-MI and plays an important role in cardiac remodeling^46^.

Daniel et al. found that MI increased the number of neutrophils and macrophages in the infarct regions 1 and 3 days post-MI in both WT and hKO mice when compared to their respective sham groups. However, there was a decrease in the number of neutrophils and macrophages in the infarct region 1 day post-MI in ATM deficient hearts. Interestingly, there was no difference between the number of inflammatory cells in WT and hKO mice 3 days post-MI. Furthermore, TGF-β levels were reduced in the infarct area of ATM deficient mice 3 days post-MI^34^. Together these results suggest that ATM deficiency results in a delayed inflammatory response early post-MI. Delayed inflammatory response along with myocyte hypertrophy and myocardial fibrosis may help explain better function during ATM deficiency 1 day post−MI. Future investigations are needed to identify the molecular signals leading to delayed inflammatory response during ATM deficiency and its long-term impact on the healing processes of the heart post-MI.

### Metabolic disorder and oxidative stress

Among the many dispositions associated with AT is the development of insulin resistance, a major characteristic of metabolic syndrome. As aforementioned, Yang and Kastan provided evidence insinuating the role of ATM in insulin-mediated protein synthesis. ATM kinase activity is activated in response to insulin, leading to subsequent ATM phosphorylation of 4EBP1 at serine 111. Consequently, 4EBP1 dissociates from eIF-4E initiating mRNA translation. Interestingly, mouse fibroblasts lacking ATM and cells from AT patients experience reduced 4EBP1/eIF-4E dissociation induced by insulin^1^. Furthermore, insulin resistance plays a role in the pathogenesis of heart failure^47^, another common feature of AT. ATM deficiency is suggested to increase the risk of dying from cardiovascular disease. ATM haploinsufficiency results in atherosclerotic lesions in apoE null mice accompanied by insulin resistance and glucose intolerance. Atm^+/−^ apoE^−/−^ mice also develop atherosclerosis coupled with hypertension, hypercholesterolemia, glucose intolerance, etc^48^. Together, these studies provide evidence for the relationship between ATM and metabolic aberrations linked to cardiovascular disease.

In addition to metabolic syndrome, ATM plays a role in oxidative stress signaling. ATM can indirectly regulate oxidative stress signaling by causing persistent double strand breaks or can act more directly by responding to irregular levels of ROS^49^. Fibroblasts from AT patients are more susceptible to hydrogen peroxide induced oxidative damage when compared to normal cells. In fact, ATM is activated in response to hydrogen peroxide and several other ROS generating agents^48^. ATM deficient cells also exhibit heightened mitochondrial dysfunction, a common characteristic of defective mitophagy^20^. Interestingly, inhibition of ATM in adult rat cardiac myocytes results in increased apoptosis coupled with an increase in ROS^38^, a phenomenon that inactivates mitophagy and activates cell death pathways if prolonged^50^. Thus, it is possible that ATM inhibition elevates apoptosis in cardiac myocytes partly via ROS-induced mitochondrial dysfunction and mitophagy impairment. Such impairment may ultimately prompt the development of heart disease. Collectively, these studies suggest that ATM may be essential in maintaining redox and mitochondrial homeostasis that can ultimately protect against cardiovascular disease.

## Conclusion

Although the role of ATM in DNA damage signaling has been extensively studied, little is known about its role in cardiac remodeling. However, recent studies have provided insight into the dynamic role ATM plays in cardiac remodeling following insults such as β-AR stimulation and MI, providing evidence that ATM modulates cardiac remodeling by affecting inflammatory response, apoptosis, fibrosis, and hypertrophy in the heart. Despite such discoveries, there are still unanswered questions regarding the exact mechanism(s) by which ATM regulates cardiac remodeling. Further investigations into the mechanism(s) by which ATM modulates functional and structural components of the failing heart may be crucial in the development of novel treatments for patients with AT.
